# Naringenin protects against acute pancreatitis-associated intestinal injury by inhibiting NLRP3 inflammasome activation *via* AhR signaling

**DOI:** 10.3389/fphar.2023.1090261

**Published:** 2023-01-13

**Authors:** Xu Yan, Tianjiao Lin, Qingyun Zhu, Yushi Zhang, Zhimin Song, Xinting Pan

**Affiliations:** The Affiliated Hospital of Qingdao University, Qingdao, China

**Keywords:** severe acute pancreatitis, intestinal injury, naringenin, AhR, NLRP3 inflammasome

## Abstract

**Background:** In this study, we examined the functions and mechanisms by which naringenin protects against SAP (severe acute pancreatitis)-related intestinal injury by modulating the AhR/NLRP3 signaling pathway.

**Material and methods:** Fifteen healthy male C57BL/6 mice were randomly divided into SAP (*n* = 12) and normal (*n* = 3) groups. Mice in the SAP group received caerulein and lipopolysaccharide intraperitoneal injections and were then randomly assigned to the SAP, NAR, CH223191, and Dexamethasone (DEX) groups. Pathological changes in the pancreatic and intestinal mucosa were observed by Hematoxylin & Eosin (H&E) staining. *In vitro*, RAW264.7 cells were exposed to lipopolysaccharide and treated with naringenin. The levels of NLRP3, AhR, IL-1β, TNF, and IL-6 in the SAP model and RAW264.7 cells were evaluated by enzyme-linked immunosorbent assay (ELISA), quantitative real-time PCR (qRT-PCR), western blot, and immunohistochemistry. The nuclear translocation of AhR was shown by immunofluorescence. AutoDockTools was used to predict the conformations of naringenin-AhR binding, and PyMol 2.4 was used to visualize the conformations.

**Results:** Mouse pancreatic and intestinal injury was alleviated by treatment with naringenin. Naringenin inhibited the activation of the NLRP3 inflammasome and inhibited damage to intestinal tight junctions. Moreover, naringenin increased AhR nuclear translocation and activated the AhR pathway.

**Conclusion:** Naringenin can reduce SAP-associated intestinal injury by inhibiting the activation of the NLRP3 inflammasome *via* the AhR signaling pathway.

## Introduction

Acute pancreatitis (AP) is the most common cause of acute abdominal disease hospital admissions (Boxhoorn et al., 2020). Gallstone and alcohol abuse are the top two risk factors that cause acute pancreatitis. The main clinical symptoms are edema, hemorrhage, and necrosis from mild to severe, which can also result in systemic inflammatory response syndrome (SIRS) and lead to multiple organ dysfunction syndrome (MODS).

Intestinal injury is secondary to severe acute pancreatitis and results in aggravation of the systemic inflammatory response, which accounts for the high mortality of AP (He et al., 2017). Liu et al. (2022) reported that inflammatory response outbreaks, oxidative stress damage, and endocrine disorders are the three main mechanisms of intestinal injury in AP. The development and prognosis of intestinal damage in AP are significantly influenced by the inflammatory response (Ge et al., 2020). Therefore, alleviation of AP-associated intestinal injury is a key therapeutic approach to severe acute pancreatitis (SAP). However, few drugs can treat AP-associated intestinal injury, and the efficiency is not satisfactory. Identifying an effective drug to inhibit the intestinal injury of acute pancreatitis is of vital importance.

The development of AP is closely related to the accumulation and infiltration of inflammatory cells and factors, according to previous research (Liu et al., 2022). AhR has gained recognition as a critical regulator of host-environment interactions in recent years, particularly for immunological and inflammatory responses (Stockinger et al., 2014; Vogel et al., 2014). The previous studies have indicated the mechanisms of AhR ligands to inhibit inflammation including Thymic Atrophy, Apoptosis, Treg Induction, MDSCs, Cytokine Suppression, and Epigenetic Changes (Cannon et al., 2021). Moreover, AhR activation in epithelia may be a crucial mechanism for controlling intestinal inflammation (Furumatsu et al., 2011).

For the host immune system to defend itself against external infections, the NLRP3 inflammasome, which belongs to the nucleotide-binding domain leucine-rich repeat family, is necessary (Kelley et al., 2019). Extensive research has been performed on NLRP3, which may have a role in several disorders, including Inflammatory bowel disease (IBD), Alzheimer’s disease, obesity, atherosclerosis, and endotoxin shock (Bauer et al., 2010; Mao et al., 2013; Abderrazak et al., 2015; Han et al., 2015). Procaspase-1 and Apoptosis-associated speck-like protein containing (ASC), which cause procaspase-1 to become active caspase-1 and mature pro-IL-1 and pro-IL-18 into biologically active and mature IL-1 and IL-18, respectively, are required for the creation of the NLRP3 inflammasome. Recently, research has indicated that the caspase-1 pathway plays a major role in SAP-associated intestinal injury involving NLRs, including NOD1, NOD2, and NLRP3 (Xu et al., 2018). What’s more, NLRP3 inflammasome activation has recently been demonstrated to be effectively suppressed by AhR, and some flavonoids are natural AhR ligands (Denison and Nagy, 2003).

Naringenin has been demonstrated to possess anti-inflammatory, organ-protective, and antioxidant activities (Kanthlal et al., 2020). A.A. Fouad et al. (2016) demonstrated that rats exposed to acute lung damage caused by lipopolysaccharide were considerably protected by naringenin. Some clinical trials also indicated that naringenin effectively inhibited the inflammatory disorders and the therapeutic effects of naringenin on primary osteoporosis, obesity and bronchial pneumonia (Jiannong et al., 2015; Namkhah et al., 2021; Yao et al., 2021). In this study, we examined the functions and mechanisms by which naringenin protects against AP-related intestinal injury by modulating the AhR/NLRP3 signaling pathway.

## Materials and methods

### Reagents and antibodies

Lipopolysaccharide and caerulein Meilinbio (Dalian, China). CH223191, dexamethasone (DEX), and naringenin were purchased from MedChemExpress (New Jersey, United States). NLRP3, AhR, ZO-1, and occludin antibodies were purchased from Abcam (Cambridge, United Kingdom). Antibodies against β-actin and histone H3 were purchased from BOSTER (Wuhan, China). Goat anti-rabbit IgG was purchased from Absin (Wuhan, China).

### Pharmacokinetic characteristics of naringenin

Oral bioavailability (OB) is considered as the most crucial component of oral medications, which may assess how well they work when disseminated to the entire body after absorption. The TCMSP database’s guidelines and earlier studies state that an OB value of less than 30% is thought to have pharmacological activity (Ru et al., 2014). Drug-likeness (DL) is a concept to determine whether novel compounds fit the criteria to become a new drug, it is based on the chemical structure of existing drugs. According to earlier study, the DL threshold value is .18 (Zhang et al., 2020). Then we used the TCMSP database to predict the absorption, distribution metabolism, and excretion (ADME) pharmacokinetic characteristics information of naringenin.

### Animal experiment

Healthy male C57BL/6 mice were obtained from the Qingdao University Laboratory Animal Center (Qingdao, China). Three mice each were randomly assigned to one of five groups: the NS group (control group), the SAP group, the NAR group, the CH223191 group, and the DEX group. CH223191 is a specific inhibitor of AhR to block the transduction of AhR. Dexamethasone (DEX), a glucocorticoid commonly used to treat AP, was used as a positive control. Before the experiment, all mice spent 2 weeks acclimating to the new environment. The mice were fasted for approximately 18 h prior to the experiment. Drinking water was available without restriction. Mice were given intraperitoneal injections of caerulein at a weight ratio of 50 μg/kg once every hour for six straight hours to induce SAP. At the same time, intraperitoneal injections of lipopolysaccharide at a weight ratio of 10 mg/kg were given to induce SAP. The same volume of ordinary saline was similarly administered to the mice in the NS group. Naringenin (50 mg/kg) was administered by intraperitoneal injections to the mice in the NAR group (Li et al., 2018). Mice in the CH223191 group received intraperitoneal injections of CH223191 (10 mg/kg) and naringenin (50 mg/kg) 30 min later (Wang et al., 2018). The DEX group of mice received an intraperitoneal injection of dexamethasone (2.5 mg/kg) (Yu et al., 2019). Twelve hours after injection, animals were euthanized, and samples were collected for further analysis.

### Cell culture, transfection and treatment

RAW264.7 cells were obtained from ATCC and cultured in DMEM (Procell, Wuhan, China) containing 10% fetal bovine serum (Procell) in an incubator at 37°C and 5% CO2.

RAW264.7 cells were seeded into 6-well plates the day before transfection at a confluency of 70%–80% for transfection experiments. After the cells had grown for 24 h, the medium was changed to serum-free medium. Then, 200 μL of DMEM (Procell) was incubated at room temperature for 20 min with 100 pmol of siAhR pool or siNC (GenePharma, Shanghai, China) and 8 μL of Lipofectamine 2000 (Invitrogen, California, United States) to facilitate complex formation. Subsequently, the cells were cultivated for 48 h at 37°C and 5% CO_2_ in an incubator. The transfection mixture was gradually added to each well. The cells were then grown in an incubator for 48 h at 37°C with 5% CO_2_.

The cells were divided into six groups: control, LPS, LPS + NAR, LPS + NAR + siAhR, LPS + NAR + siNC, and LPS + NAR + CH223191. The dosages administered were 1 μg/mL for LPS, 1 μM for CH223191 (Wang et al., 2018), and 50 μM for naringenin. CH223191 was added 30 min after naringenin was added. The treatments were conducted 24 h later.

### CCK8 cell viability assay

The CCK8 assay was used to assess the viability of RAW264.7 cells. RAW264.7 cells were seeded into 96-well plates and incubated with naringenin (10, 20, 40, 50, and 80 μM) for 24 h. Then, each well received 10 μL of CCK-8 for 1 h, and the absorbance was measured at 450 nm. LPS (1 μg/mL) was added to the cells to establish inflammatory model, and gradient concentrations of naringenin (20 μM, 40 μM, and 50 μM) were treated with the cells for 12 and 24 h. Consequently, the viability of RAW264.7 was detected by CCK8 assay to find a concentration of naringenin that significantly inhibited the proliferation of inflammatory cells.

### Enzyme-linked immunosorbent assay (ELISA)

The levels of tumor necrosis factor (TNF), interleukin 1 beta (IL-1β) and interleukin 6 (IL-6) in the serum of mice and cell culture supernatant were detected using the corresponding ELISA kits (ABclonal, Wuhan, China) according to the kit instructions.

### Hematoxylin & Eosin (H&E) staining

The intestines collected from mice were dehydrated using a gradient of ethanol after being fixed in 4% paraformaldehyde for 24 h at 4°C. The samples were subsequently cut into 5-μm-thick slices and fixed in paraffin. Following the recommended procedures, the sections were cleaned, rehydrated, and stained with hematoxylin and eosin (Solarbio, Beijing, China). The staining was observed under a microscope and captured in a ×200 magnification image. Pancreatitis histopathological changes were assed using the standards put forth by Schmidt et al. (1992). The grades of ileum damage were determined by Chiu’s description of the classification of gut injury (Chiu et al., 1970). There are five grades from mild to severe forms: Grade 0, normal mucosal villi; grade 1, Gruenhagen’s space development beneath the epithelium, typically at the villus’s tip; frequently accompanied by capillary congestion; grade 2, Extension of the subepithelial space with moderate lifting of epithelial layer from the lamina propria; grade 3, massive epithelial lifting down the sides of villi, possibly with a few denuded tips; grade 4, denuded villi with the lamina propria and dilated capillaries exposed, possibly with increased cellularity of the lamina propria; and grade 5, digestion and disintegration of the lamina propria, hemorrhage and ulceration.

### Immunohistochemical staining

The paraffin slices underwent standard dewaxing and hydration procedures. In sodium citrate buffer, 3% H_2_O_2_ was used to block non-specific antibody binding sites after the antigen was microwave-recovered. Then, the sections were incubated at 4°C overnight with the corresponding primary antibody (1:200) and goat-anti-rabbit IgG that had been HRP-labeled for 1 h at 37°C. Hematoxylin was used in conjunction with DAB to visualize the staining. The sections were examined at ×400 magnification and captured with a camera using a microscope.

### Immunofluorescence assay

RAW264.7 cells were cultured on coverslips, and bovine serum albumin (BSA) was used to block the tissue after it had been fixed with 4% paraformaldehyde for 30 min and permeabilized with .5% Triton X-100 for 15 min. The cells were then treated with an Alexa Fluor-labeled secondary antibody (1:100) for 2 hours after being immunostained with an AhR antibody (1:100) overnight at 4°C. The images were finally obtained using an Olympus 1X51 microscope.

### Quantitative real-time PCR

Total RNA was extracted from the ileum tissue and cell samples using TRIzol reagent. An Evo M-MLV RT Mix Kit with gDNA clean for qPCR (AG, Changsha, China) was used to create cDNA from 2 μg of total RNA. Real-time PCR was carried out as instructed by the SYBR Green kit.

The 2^−ΔΔCT^ method was used to quantitatively evaluate the level of mRNA expression. The primer sequences are shown in Table 1.

**TABLE 1:** Primer Sequences for qRT -PCR.

Primer	Sequence
IL-1β	F: GCC​ACC​TTT​TGA​CAG​TGA​TGA​G
	R: ATG​TGC​TGC​TGC​GAG​ATT​TG
IL-6	F: GTC​CTT​CCT​ACC​CCA​ATT​TCC​A
	R: TAA​CGC​ACT​AGG​TTT​GCC​GA
TNF	F: GTA​GCC​CAC​GTC​GTA​GCA​AA
	R: ACA​AGG​TAC​AAC​CCA​TCG​GC
NLRP3	F: GCT​GTG​TGA​GGC​ACT​CCA​G
	R: GGA​GAT​GTC​GAA​GCA​GCA​TT
AhR	F: TTC​TTA​GGC​TCA​GCG​TCA​GCT​A
	R: GCA​AAT​CCT​GCC​AGT​CTC​TGA​T
ZO-1	F: GAT​AGT​TTG​GCA​GCA​AGA​GAT​GGT​A
	R: AGG​TCA​GGG​ACG​TTC​AGT​AAG​GTA​G
Occludin	F: CCT​TCT​GCT​TCA​TCG​CTT​CCT​TA
	R: CGT​CGG​GTT​CAC​TCC​CAT​TAT

### Western blot

Protein was isolated from intestinal tissue and cells, and the concentration of the protein was measured using a BCA quantitative kit (Meilinbio, Dalian, China). Sodium dodecyl sulfate (SDS)-polyacrylamide gel electrophoresis (PAGE) was used to load the protein samples and transfer the protein samples onto polyvinylidene fluoride (PVDF) membranes. The membranes were incubated with primary antibody at 4°C overnight after being blocked with 5% skim milk for 2 h. Following a TBST buffer wash, the PVDF membranes were incubated with IgG-HRP secondary antibody (1:5,000) for 1 h. Electrochemiluminescence substrate (Meilinbio) was used to visualize the proteins, and ImageJ was used to assess the gray value.

### Molecular docking

Molecular docking is able to anticipate the binding mechanism and affinity between small molecule ligands and biological macromolecular receptors by simulating their interaction (Udrea et al., 2021). The crystal structure of AhR was obtained from the PDB website, and the 3D structure of naringenin was retrieved from the PubChem database. Naringenin was the ligand, and AhR was the receptor. The receptor was added to AutoDock 4.2.6 and PyMol 2.4 for the removal of heteroatoms and water molecules as well as the addition of charges and hydrogen atoms. Then, AutoDockTools was used to predict the conformations of ligand‒receptor binding, and PyMol 2.4 was used to visualize the conformations.

### Statistical analysis

GraphPad Prism 8.0.2 was used to perform the statistical analyses. To assess the significance of the variations between the groups, a standard one-way analysis of variance was performed. The data are expressed as the mean ± SD. Results were considered significant when *p* < .05.

## Results

### Naringenin reduces pancreatic and intestinal injury in mice with SAP

**FIGURE 1:**
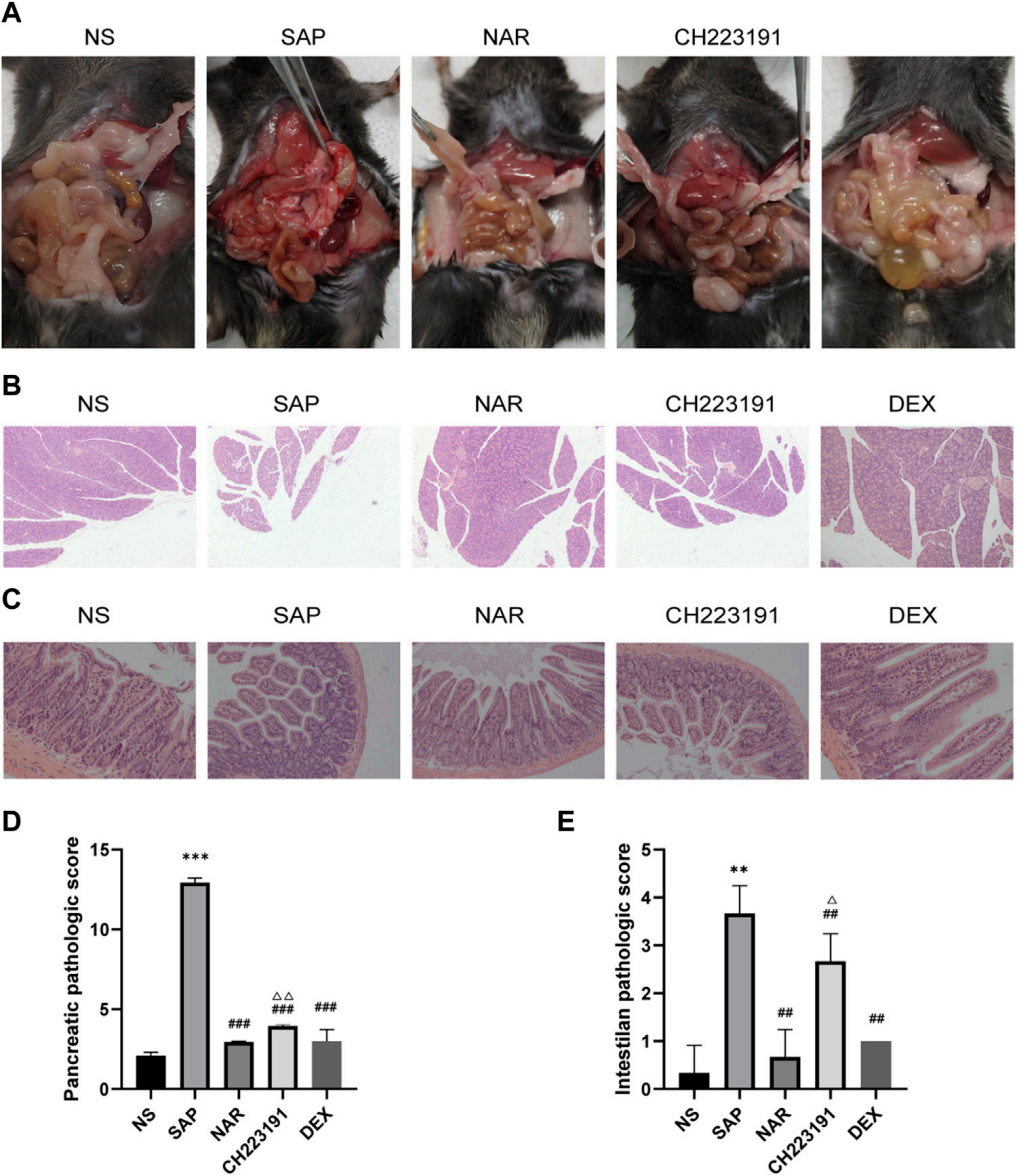
Naringenin attenuates pancreatic and intestinal injury in mice with SAP. **(A)** Representative photos of pancreas and intestinal damage in the NS group (*n* = 3), SAP group (*n* = 3), NAR group (*n* = 3), CH223191 (*n* = 3) and DEX group (*n* = 3). **(B)** Pathological changes in the pancreas in mice (H&E staining 200×). **(C)** Pathological changes in the intestine in mice (H&E staining 200×). **(D)** Pancreatic inflammation scores of each group. **(E)** Intestine inflammation scores of each group. **p < .001, ***p < .0001 vs. NS; ##*p* < .001, ###*p* < .0001 vs. SAP; ΔP<.01, ΔΔP<.001 vs. NAR.

First, the Pharmacokinetic Characteristics of Naringenin were showed in Table 2. Then, we assessed the effects of naringenin on SAP mouse pancreas. Pancreatic H&E staining of the SAP model mice revealed a clear focal necrotic region, lobular structural destruction, inflammatory cell infiltration, and hemorrhage (Figures 1A, B), and there were significant differences between these groups in the pancreas pathological scores (Figure 1D). In addition, the pancreatic damage was effectively reduced by naringenin administration. The impact of naringenin on pancreatic damage was eliminated by CH223191.

**TABLE 2:** The pharmacokinetic characteristics of naringenin.

Molecule id	Molecule name	OB (%)	Caco-2	BBB	DL	HL
MOL004328	naringenin	59.29	.28	−.37	.21	16.98

OB, oral bioavailability; Caco-2, Caco-2 permeability; BBB, blood-brain barrier; DL, drug-likeness; HL, half-life.

Next, we investigated how naringenin affected intestinal injury. H&E staining of the SAP group and CH223191 group showed intestinal villus rupture and mucosal injury, with a clearly increased inflammation score (Figure 1E). Naringenin effectively alleviated the pathological damage (Figure 1C).

### Naringenin inhibits intestinal inflammation in mice with SAP

**FIGURE 2:**
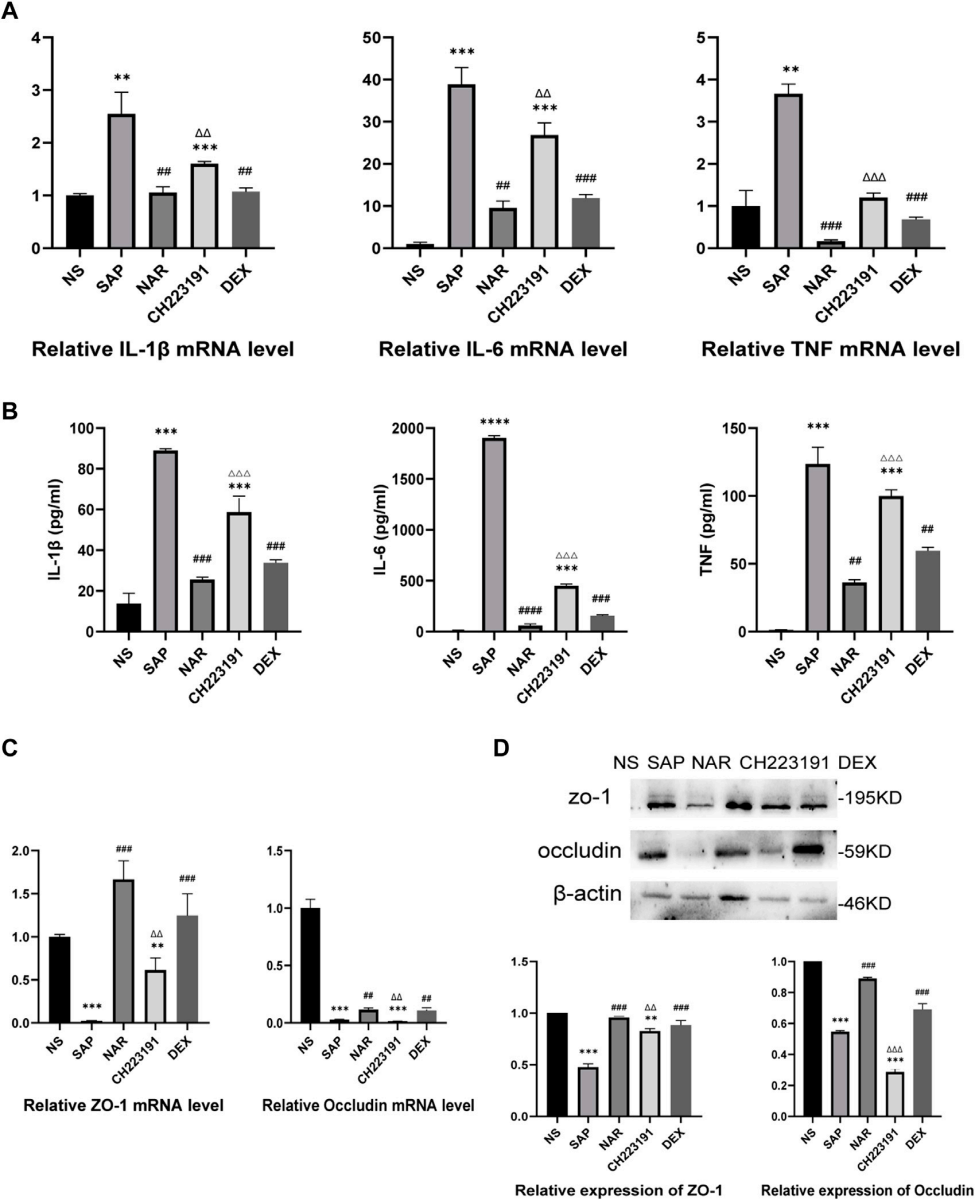
Naringenin inhibits intestinal inflammation in mice with SAP. **(A)** Relative expression levels of IL1β, IL-6, and TNF mRNA in the intestine as analyzed by real-time PCR. **(B)** The levels of IL1β, IL-6, and TNF-α in the intestine as detected by ELISA. **(C)** Relative expression levels of occludin and ZO-1 mRNA in the intestine as analyzed by real-time PCR. **(D)** Western blot analysis of occludin and ZO-1 expression in the intestine of each group and corresponding grayscale statistics. **p < .001, ***p < .0001 vs. NS, ##*p* < .001, ###*p* < .0001 vs. SAP, ΔΔP<.001, ΔΔΔP<.0001 vs. NAR.

The expression of inflammatory cytokines, including IL-1β, IL-6, and TNF, was increased in the SAP group compared with that in the NS group (Figures 2A, B). The NS and NAR groups showed less secretion of these cytokines. In addition, qRT‒PCR (Figure 2C) and western blot (Figure 2D) analyses revealed low levels of occludin and ZO-1 expression in the SAP group, while naringenin treatment inhibited the decrease in occludin and ZO-1. CH223191 consistently reversed the effect of naringenin on intestinal inflammation.

### Naringenin inhibits activation of the NLRP3 inflammasome

**FIGURE 3:**
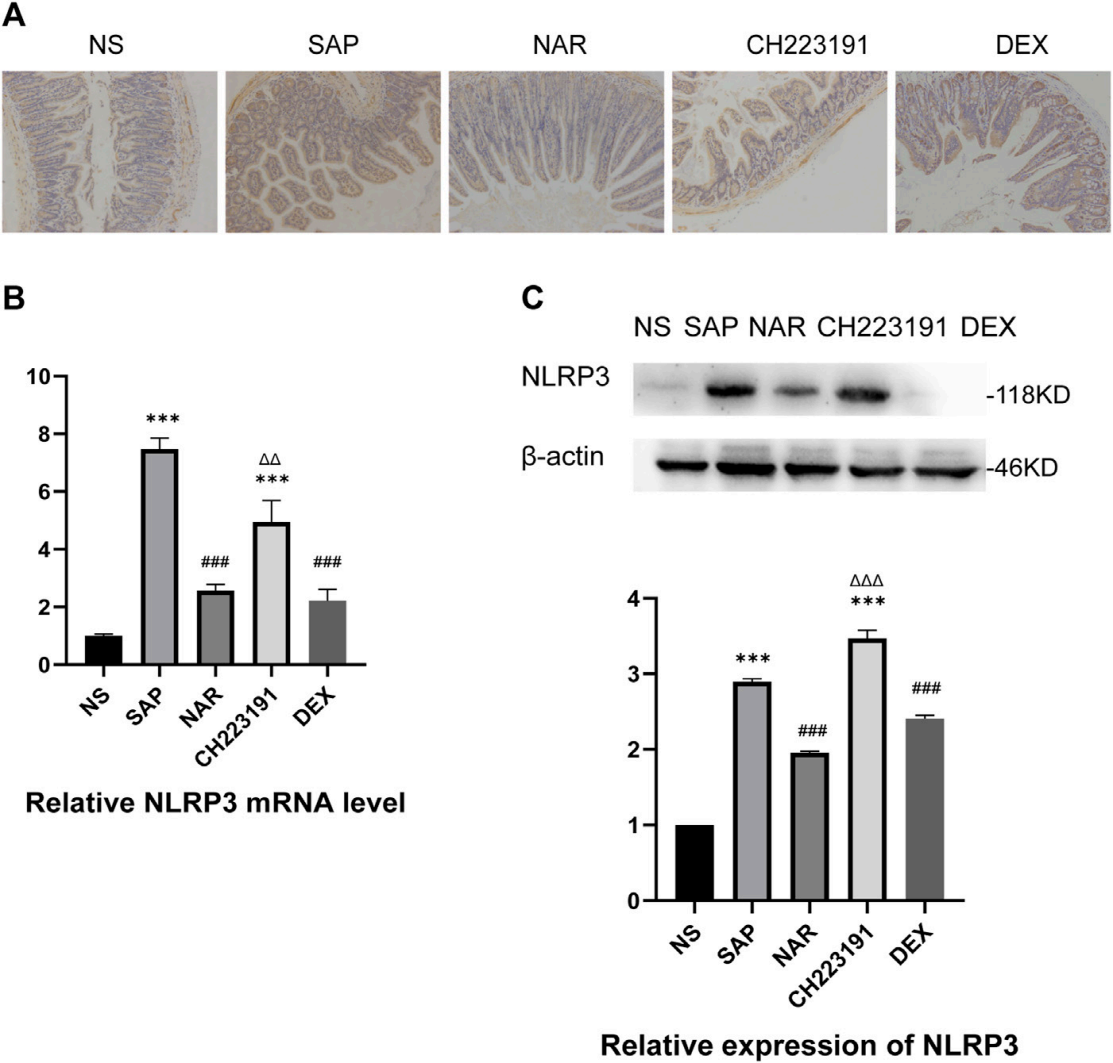
Naringenin inhibits activation of the NLRP3 inflammasome. **(A)** Immunohistochemical staining was performed on ileum sections to detect NLRP3. **(B)** Relative expression levels of NLRP3 mRNA in the intestine, as analyzed by real-time PCR. **(C)** Western blot analysis of NLRP3 expression in the intestine of each group and corresponding grayscale statistics. ***p < .0001 vs. NS; ###*p* < .0001 vs. SAP, ΔΔP.

It is generally known that NLRP3 forms the NLRP3 inflammasome with ASC and caspase-1, which leads to inflammation by activating and producing IL-1β and IL-18 (Udrea et al., 2021). Immunohistochemistry, qRT‒PCR, and western blot analyses (Figure 3) showed significantly increased expression of NLRP3 in intestinal tissue in the SAP group, while the NAR group exhibited reduced levels of NLRP3. Similarly, CH223191 reversed the effect of naringenin.

### Naringenin activates AhR *in vivo* and molecular docking with AhR

**FIGURE 4:**
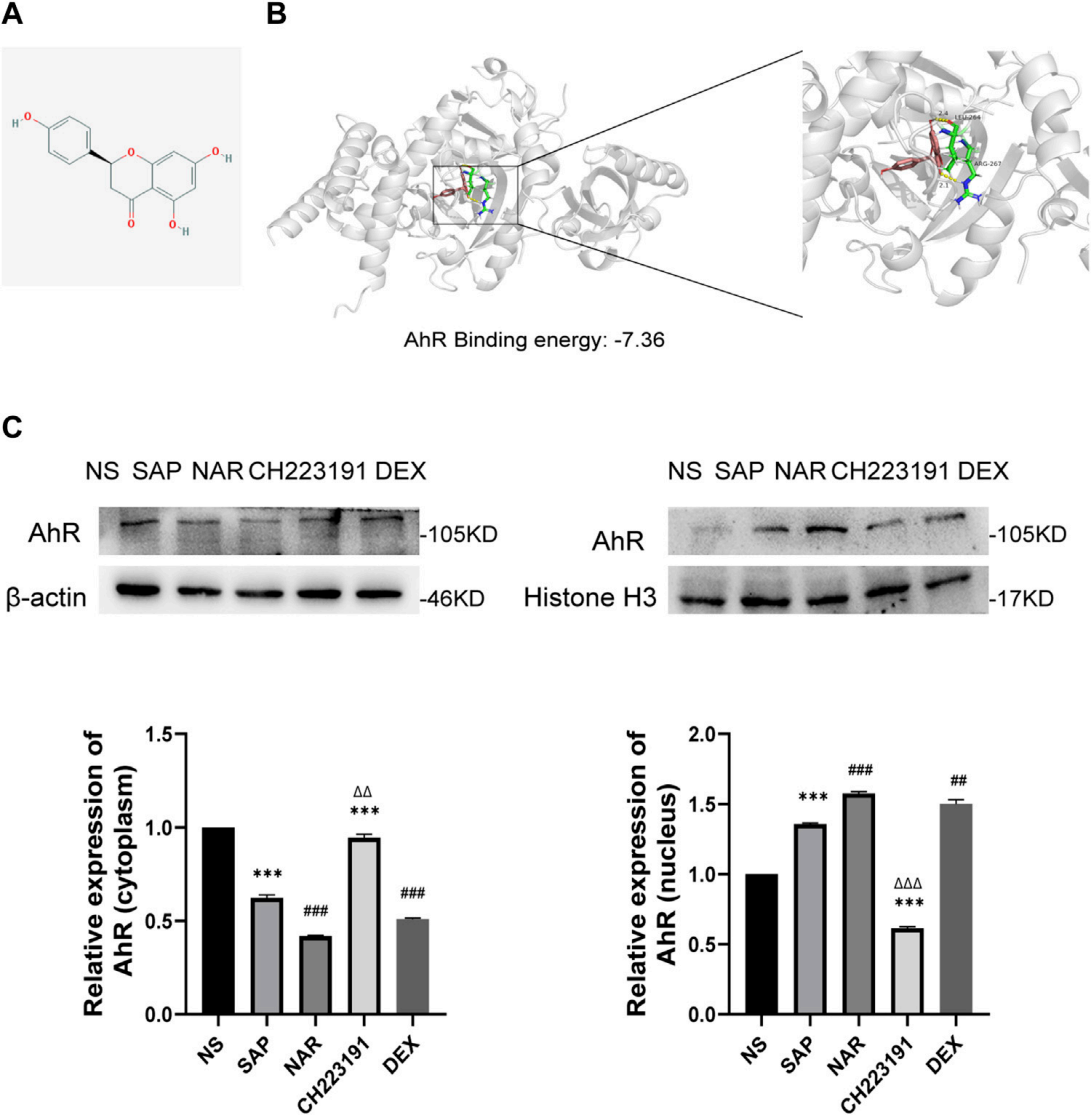
Naringenin activates AhR *in vivo* and the molecular docking with AhR. **(A)** Structure of naringenin. **(B)** 3D docking conformation of naringenin with AhR. **(C)** Western blot analysis of AhR expression in the cytoplasm and nucleus of each group and corresponding grayscale statistics, *n* = 3. ***p < .0001 vs. NS, ##*p* < .001, ###*p* < .0001 vs. SAP, ΔΔP<.01, ΔΔΔP<.001 vs. NAR.

We used AutoDock 4.2.6 for molecular docking, and the binding energy between AhR and naringenin was −7.36 kcal/mol. According to earlier research, a binding affinity of < −4.25 kcal/mol signifies that the two molecules could connect, <−5 kcal/mol indicates good binding, and <−7.0 kcal/mol suggests significant binding activity (Grosdidier et al., 2011). The docking result is displayed in a 3D graph (Figures 4A, B), and there were two hydrogen bonds (the yellow dotted lines represent hydrogen bonds) between AhR and naringenin. As shown, naringenin bound to the LEU-264 and ARG-267 binding sites of AhR. Naringenin administration promoted AhR nuclear translocation and increased its expression, as demonstrated by western blots, while CH223191 blocked the effect of naringenin on the AhR signaling pathway (Figure 4C).

### Naringenin inhibits RAW264.7 cell inflammation by AhR and NLRP3 inflammasome activation

**FIGURE 5:**
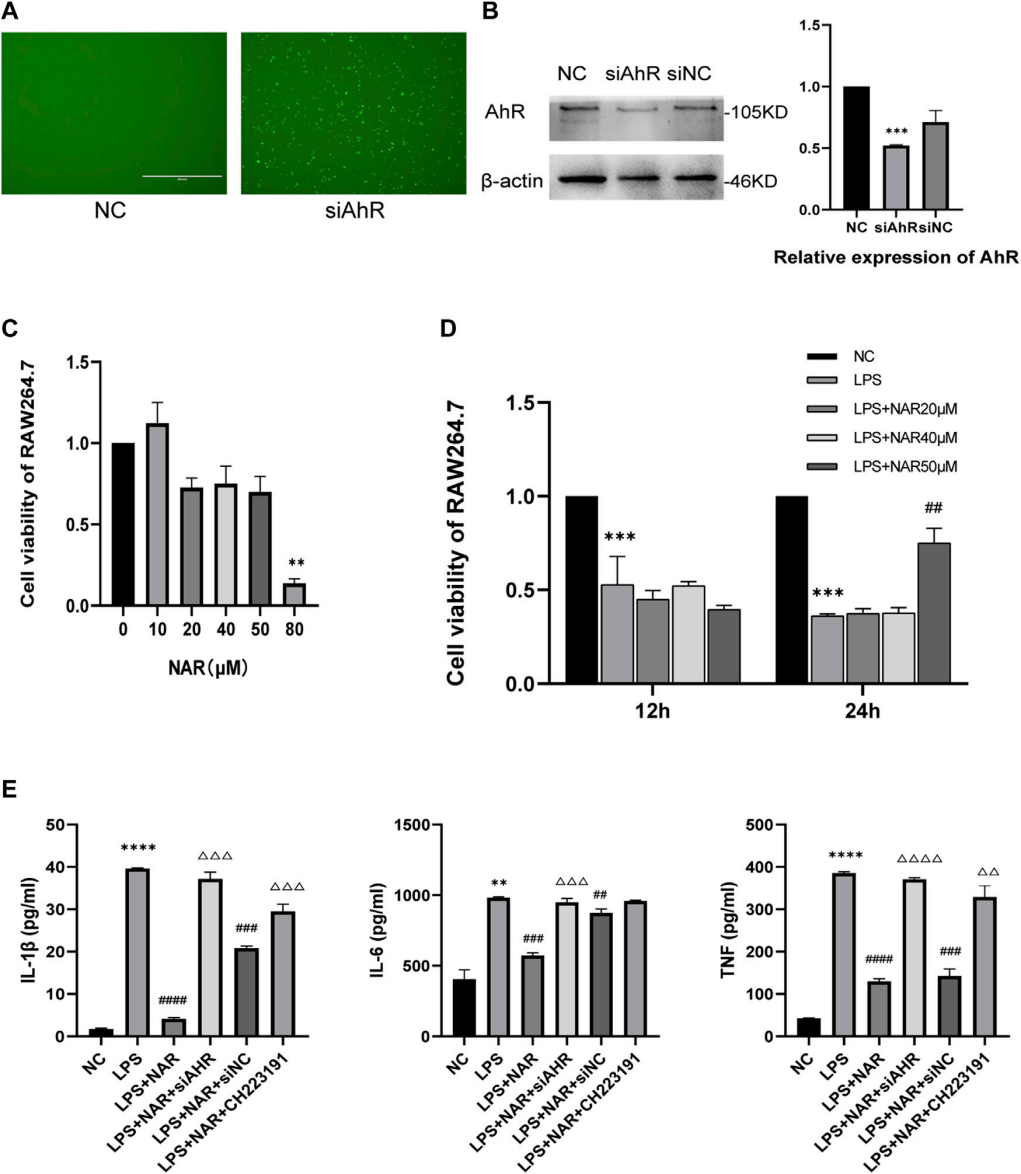
Naringenin inhibits RAW264.7 cell inflammation by AhR and NLRP3 inflammasome activation. **(A)** RAW264.7 cell images obtained using a microscope after fluorescence staining and siRNA transfection. **(B)** Western blot analysis of AhR expression in the cytoplasm and corresponding grayscale statistics. **(C)** CCK8 analysis of cell viability of Raw264.7 cells after treatment with different concentrations of naringenin (10 μM, 20 μM, 40 μM, 50 μM and 80 μM) for 24 h **(D)** CCK8 analysis of cell viability of Raw264.7 after treatment with LPS (1 μg/mL) and different concentrations of naringenin (20 μM, 40 μM and 50 μM) for 12 and 24 h. **(E)** The levels of IL-1β, IL-6 and TNF in the cell supernatants of each group as detected by ELISA. **p < .01, ***p < .001 vs. NC group, ##*p* < .05, ###*p* < .01, ####*p* < .001 vs. LPS group, ΔΔP<.05, ΔΔΔP<.01, ΔΔΔΔP<.001 vs. LPS + NAR group.

We created an *in vitro* inflammatory cell model to better evaluate the therapeutic effect of naringenin on AP-related intestinal injury. An AhR-knockdown cell model was successfully created, as shown in Figures 5A, B. And the CCK8 assay (Figures 5C, D) showed that naringenin at a concentration of 50 μM significantly inhibited the proliferation of inflammatory cells. According to the ELISA results (Figure 5E), the expression of IL-1β, IL-6, and TNF in LPS-treated RAW264.7 cells was significantly upregulated, while naringenin reduced the expression of IL-1β, IL-6, and TNF. In addition, the effects of naringenin were eliminated by both CH223191 and AhR siRNA.

### Naringenin Inhibits NLRP3 inflammasome activation

**FIGURE 6:**
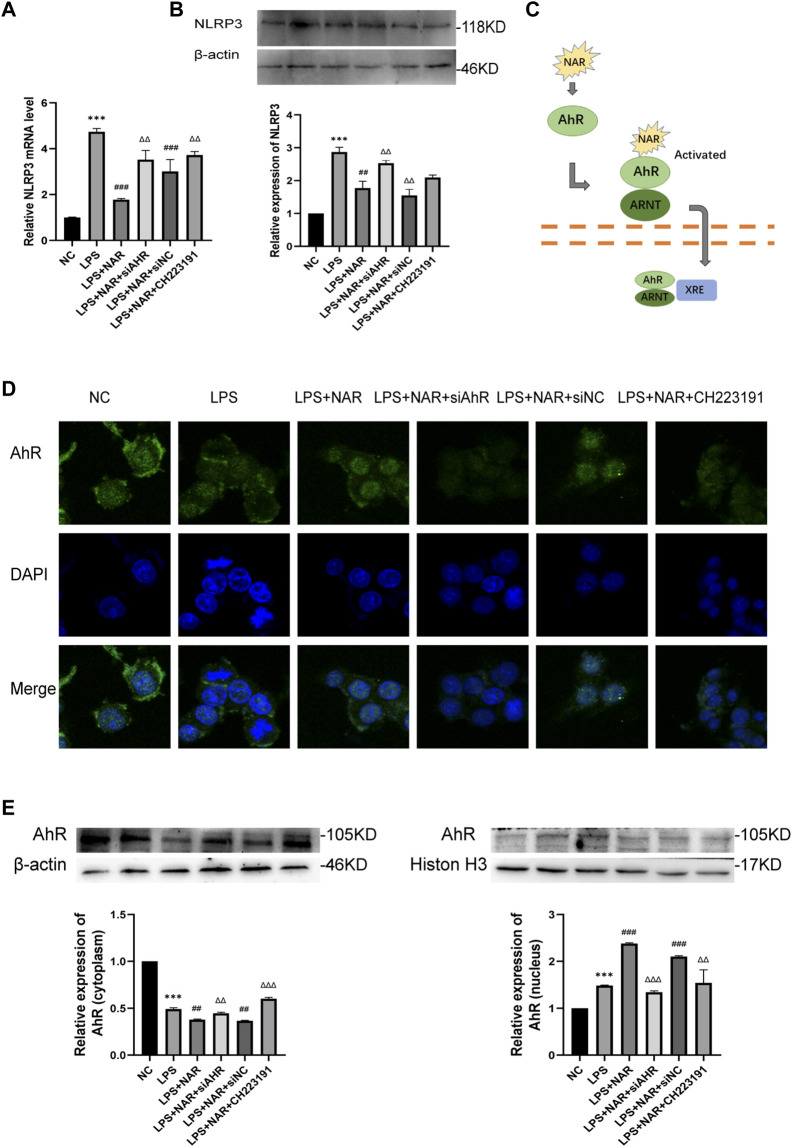
Naringenin Activates AhR signaling *in vitro*. **(A)** Relative expression levels of NLRP3 mRNA in RAW264.7 cells of the NC group, LPS group, LPS + NAR group, LPS + NAR + siAhR group, LPS + NAR + siNC group and LPS + NAR + CH223191 group as analyzed by qRT‒PCR. **(B)** Western blot analysis and corresponding grayscale statistics of NLRP3 expression in each group. **(C)** Scheme of naringenin-induced activation of AhR signaling. **(D)** AhR nuclear translocation in RAW264.7 cells as detected by immunofluorescence in the NC group, LPS group, LPS + NAR group, LPS + NAR + siAhR group, LPS + NAR + siNC group and LPS + NAR + CH223191 group. **(E)** Western blot analysis of AhR expression in the cytoplasm and nucleus of each group and corresponding grayscale statistics. ***p < .0001 vs. NC group, ##*p* < .001, ###*p* < .0001 vs. LPS group, ΔΔP<.001, ΔΔΔP<.0001 vs. LPS + NAR group.

Western blot and qRT‒PCR analyses (Figures 6A, B) revealed that NLRP3 expression was considerably upregulated in LPS-treated RAW264.7 cells and downregulated in response to naringenin treatment. Similarly, CH223191 and AhR siRNA reversed the effect of naringenin.

### Naringenin activates AhR *in vitro*

AhR nuclear translocation was facilitated, and the expression of AhR was increased by naringenin therapy, as seen by immunofluorescence staining (Figures 6C, D). In contrast, the effect of naringenin on the AhR pathway was reversed by either CH223191 or AhR siRNA (Figure 6E).

## Discussion

Severe acute pancreatitis is one of the most common abdominal disorders and has a high mortality rate (Norman, 1998). The infiltration of macrophages and leukocytes and the activation of inflammatory cytokines lead to the generation of oxygen-free radicals and trigger systemic inflammatory cascades that damage the kidneys, lungs, and intestines and finally cause the high mortality of SAP. The villi fall off and shorten in response to intestinal inflammation when SAP occurs. Subsequently, the intestinal mucosa shrinks, and the normal flora is out of balance. All of these factors contribute to the development of intestinal ectopic bacterial toxins. Intestinal pathogenic alterations, such as intestinal barrier damage and microbiota changes, may exacerbate the symptoms of severe acute pancreatitis (Ammori et al., 1999).

Naringenin has been shown in a previous study to be a candidate for the treatment of SAP, although the underlying processes by which naringenin carries out its pharmacological activities still need further research (Li et al., 2018). The goal of the current investigation was to determine how naringenin affects the AhR/NLRP3 signaling pathway in SAP-related intestinal injury.

By injecting caerulein and lipopolysaccharide, we successfully established a mouse model of AP in this study, and a clear intestinal injury was produced. The intestinal barrier is composed of a mechanical barrier, an immunological barrier, a chemical barrier, and a biological barrier. The mechanical barrier has a substantial impact on intestinal function, and the alteration of intestinal mucosa tight junctions in SAP is the primary cause of increased intestinal permeability. It is widely recognized that inflammatory cytokines, including TNF, IL-1β, and IL-6, can lead to damage to the intestinal barrier. IL-1β can accelerate the course of intestinal injury by stimulating monocytes and macrophages (Ge et al., 2020), while TNF and IL-6 lead to severe apoptosis of intestinal mucosa cells (Sun et al., 1999). Moreover, occludin and ZO-1 are molecules widely linked with tight junctions of the intestinal mucosa. Our findings showed that naringenin considerably reduced the expression of inflammatory cytokines and the number of leukocytes, demonstrating that naringenin dramatically reduces pancreatic and intestinal inflammation. Our data demonstrated that naringenin therapy inhibited the destruction of ZO-1 and occludin and relieved the intestinal injury caused by SAP.

When infection and cellular injury occur, the NLRP3 inflammasome can activate caspase-1 and then induce the activation of the inflammatory cytokines IL-1β and IL-18. We observed that NLRP3 expression levels were all considerably reduced after naringenin treatment. Pyroptosis, which mostly relies on caspase-1 activation, is considered to play an important role in intestinal injury and lead to the release of inflammatory cytokines, including IL-1β and IL18 (Ning et al., 2017). Our previous research demonstrated that SAP-associated intestinal injury is mainly caused by triggering NLRP3-dependent pyroptosis. The intestinal injury caused by SAP and pancreatic damage can be alleviated by downregulating the main executor of pyroptosis, GSDMD (Lin et al., 2021). Therefore, SAP-associated intestinal injury may be inhibited by naringenin through the suppression of pyroptosis, though this hypothesis still requires more evidence.

To investigate the upstream regulators of the NLRP3 inflammasome, we concentrated on AhR, as it has become a key therapeutic target for many disorders. Generally, AhR is considered a part of the basic helix-loop-helix/Per-Arnt-Sim superfamily and bound to several cochaperones in an inactive form in the cytosol (Wang et al., 2018). After ligand binding, the cochaperones release AhR, which is then carried into the nucleus, where it heterodimerizes with the nuclear translocator of the aryl hydrocarbon receptor (ARNT) (Neavin et al., 2018; Modoux et al., 2022; Zhang et al., 2022). According to previous research, AhR is a possible target for treating disorders with uncontrolled inflammasome activation because it inhibits the transcription of the NLRP3 inflammasome as a negative regulator of NLRP3 inflammasome activity (Huai et al., 2014). AhR is broadly expressed throughout the intestinal epithelium. Tight junctions, which are damaged in chronic inflammatory gut disorders, are likewise regulated by AhR (Stockinger et al., 2021). However, it is still uncertain whether naringenin influences AhR signaling in SAP and whether inhibited AhR expression can alleviate intestinal injury. In the present study, we hypothesized that naringenin inhibited activation of the NLRP3 inflammasome *via* the AhR signaling pathway. We used CH223191 and the siAhR pool to inhibit the expression of AhR to further examine this hypothesis. CH223191 has been introduced as a ligand-selective antagonist of AhR by blocking the endogenous ligand FICZ (Mohammadi-Bardbori et al., 2019). Our findings demonstrated that the therapeutic effect of naringenin on pancreatic and intestinal damage and inflammation was reversed by CH223191. Moreover, the impact of naringenin on the activation of the NLRP3 inflammasome was affected by siAhR and CH223191. The data showed that naringenin prevented SAP-associated intestinal injury by blocking the activation of the NLRP3 inflammasome, which is specifically activated by the AhR signaling pathway.

## Conclusion

In conclusion, this research suggests that naringenin prevents SAP-related intestinal injury by decreasing NLRP3 inflammasome activation, which is partially mediated by the AhR pathway. AhR may become a promising therapeutic target for naringenin treatment of SAP-induced intestinal injury.

## Data Availability

The datasets presented in this study can be found in online repositories. The names of the repository/repositories and accession number(s) can be found in the article/Supplementary Material.

## Abbreviations

AhR, Aryl hydrocarbon receptor; AP, Acute pancreatitis; ASC, Apoptosis-associated speck-like protein containing; BSA, Bovine serum albumin; DEX, Dexamethasone; IBD, Inflammatory bowel disease; LPS, Lipopolysaccharide; MODS, Multiple organ dysfunction syndrome; NAR, Naringenin; NLRP3, NOD-like receptor thermal protein domain associated protein 3; NLRs, NOD-like receptors; NOD1, Recombinant Nucleotide Binding Oligomerization Domain Containing Protein 1; NOD2, Recombinant Nucleotide Binding Oligomerization Domain Containing Protein 2; IL-1β, interleukin 1 beta; IL-6, interleukin 6; pro-IL-1β, pro interleukin 1 beta; pro-IL-18, pro interleukin 18; PVDF, Polyvinylidene fluoride; RNT, Nuclear translocator of the aryl hydrocarbon receptor; SAP, Severe acute pancreatitis; SIRS, Systemic inflammatory response syndrome; SDS-PAGE, Sodium dodecyl sulfate -polyacrylamide gel electrophoresis.
