# Corrigendum: Naringenin protects against acute pancreatitis-associated intestinal injury by inhibiting NLRP3 inflammasome activation via AhR signaling

**DOI:** 10.3389/fphar.2023.1295494

**Published:** 2023-10-26

**Authors:** Xu Yan, Tianjiao Lin, Qingyun Zhu, Yushi Zhang, Zhimin Song, Xinting Pan

**Affiliations:** The Affiliated Hospital of Qingdao University, Qingdao, China

**Keywords:** severe acute pancreatitis, intestinal injury, naringenin, AhR, NLRP3 inflammasome

In the published article, there was an error in Figure 3 as published. The image of the DEX group in Figure 3A was inadvertently misused during the final assembly of Figure 3. The corrected Figure 3 and its caption are provided herein.

**FIGURE 3:**
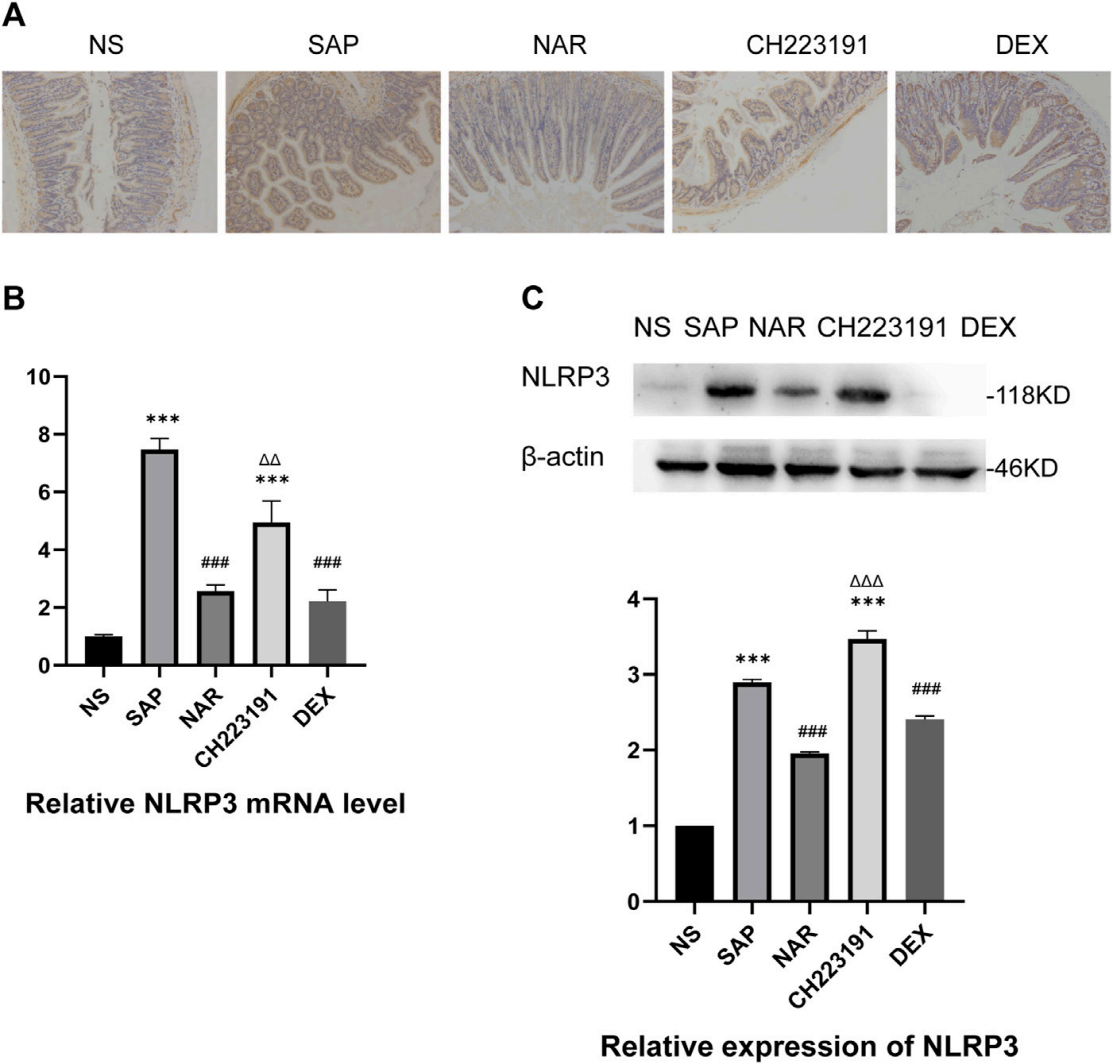
Naringenin inhibits activation of the NLRP3 inflammasome. **(A)** Immunohistochemical staining was performed on ileum sections to detect NLRP3. **(B)** Relative expression levels of NLRP3 mRNA in the intestine, as analyzed by real-time PCR. **(C)** Western blot analysis of NLRP3 expression in the intestine of each group and corresponding grayscale statistics. ***p < .0001 vs. NS; ###*p* < .0001 vs. SAP, ΔΔP.

The authors apologize for this error and state that this does not change the scientific conclusions of the article in any way. The original article has been updated.

